# Pediatric Emergency Medicine Fellow Training for Bedside Teaching: A National Survey of Pediatric Emergency Medicine Fellows and Program Directors

**DOI:** 10.7759/cureus.48652

**Published:** 2023-11-11

**Authors:** David B Kling, Sara Helwig, Maya S Iyer

**Affiliations:** 1 Pediatric Emergency Medicine, Nationwide Children’s Hospital, Columbus, USA; 2 Pediatric Emergency Medicine, Abigail Wexner Research Institute, Columbus, USA; 3 Pediatric Emergency Medicine and Biostatics, Nationwide Children’s Hospital, Columbus, USA; 4 Pediatrics, The Ohio State University College of Medicine, Columbus, USA

**Keywords:** pediatric fellows, medical education, pediatric emergency medicine, curriculum development, bedside teaching

## Abstract

Background

The pediatric emergency department (PED) is a unique fast-paced learning environment. Most pediatric emergency medicine (PEM) physicians work in academic centers and therefore require specific bedside teaching skills to improve and enhance learners’ experiences, yet there is no standardized training in bedside teaching for PEM fellows. We aimed to (1) evaluate whether PEM fellowship programs train their fellows to become bedside teachers through a dedicated curriculum and (2) determine how these programs measure curricular effectiveness.

Methods

In 2022, we developed and disseminated two separate electronic surveys to PEM fellows and PEM fellowship program directors (PDs) and associate program directors (APDs) through the American Academy of Pediatrics’ Section of Emergency Medicine PEM PD Survey Committee.

Results

One-third of PEM fellows (32.7%, n=69/211) and PEM PDs/APDs (32.5%, n=26/80) completed the surveys. The majority of fellows (59.4%, n=41/69) and PDs/APDs (76.9%, n=20/26) reported that their programs did not have a formal bedside teaching curriculum. Of the respondents, 81.2% (n=56/69) of fellows and 100% of PDs/APDs believed that their programs prepared fellows to be bedside teachers (p-value=0.04). Most fellows (75.4%, n=52/69) expressed interest in working at an academic medical center, with 19.2% (n=10/52) feeling unprepared to be bedside teachers upon completion of the fellowship. Additionally, 19.2% (n=5/26) PDs/APDs, evaluated their bedside teaching curriculum effectiveness. In addition, the majority of fellows (60.9%, n=42/69) and PDs/APDs (61.5%, n=16/26) described clinical expectations and competing fellowship priorities, respectively, as major barriers to learning bedside teaching. Most PDs/APDs (53.8%, n=14/26) stated that they were uncertain on how to create a bedside teaching curriculum.

Conclusions

While the majority of PEM fellows plan to be academicians, where they will be expected to teach a variety of learners at the bedside, most PEM fellowship programs do not have dedicated bedside teaching curricula. There is a compelling need to investigate how to encourage and implement bedside teaching education into PEM fellowship curricula.

## Introduction

With its high volumes, rapid patient turnover, and a varied, yet often critically ill patient population, the pediatric emergency department (PED) provides a distinctive learning environment for medical trainees [1]. Most practicing pediatric emergency medicine (PEM) physicians primarily work at academic institutions, where they have an important role in educating a variety of trainees [2]. However, trainees sometimes believe that their education is negatively affected by the controlled chaos of the PED, while attending physicians also think clinical responsibilities limit their time to teach [3,4]. These findings suggest physicians working with learners in the PED must be facile and adaptive bedside teachers.

Bedside teaching is defined by Aldeen and Gisondi as a “valuable instructional method that facilitates the development of history and physical examination skills, the modeling of professional behaviors, and the direct observation of learners” [5]. The teacher/educator provides learning points and direct feedback, and the learner is a medical student, resident, fellow, or faculty member. Governing medical bodies highlight the importance of teaching in the clinical environment. The inclusion of teaching benchmarks in the Accreditation Council for Graduate Medical Education (ACGME) Milestone Project highlights the importance of training fellows to be effective teachers [6-8]. Several medical specialties have dedicated educator curricula or pathways to train students, residents, and fellows [9-13]. Fellowship training provides an important opportunity to hone the teaching skills of future clinician educators, and fellows believe that such training is crucial to their future careers [2,14].

To date, little is known about how PEM fellows are trained to become bedside teachers. We aimed to 1) assess whether PEM fellowship programs train their fellows to become bedside teachers through a dedicated curriculum and 2) determine how these programs measure curricular effectiveness.

This article was previously presented as a poster abstract at the 2023 Pediatric Academic Society Annual Scientific Meeting on April 28, 2023.

## Materials and methods

Study design

We surveyed PEM fellows and PEM fellowship program directors (PDs)/associate program directors (APDs) from May to June 2022 to understand how bedside teaching is taught at their respective PEM fellowship programs. The survey was open for five weeks, and nonrespondents received two reminders. The study was approved by the home institution’s Internal Review Board with the ID# STUDY00002518.

Participants

Through a comprehensive review of the PEM PD listserv, electronic mailing list, and a search of the American Medical Association Fellowship and Residency Electronic Interactive Database (FREIDA) search engine, we identified 80 PEM fellowship programs. Upon reviewing the individual websites of these programs from the aforementioned list, we determined that during the study period, there were 620 active PEM fellows, 82 PDs, and 69 APDs [15,16]. All active PEM fellows and PDs/APDs were eligible to complete the survey. 

Survey development and dissemination

**Figure 1:**
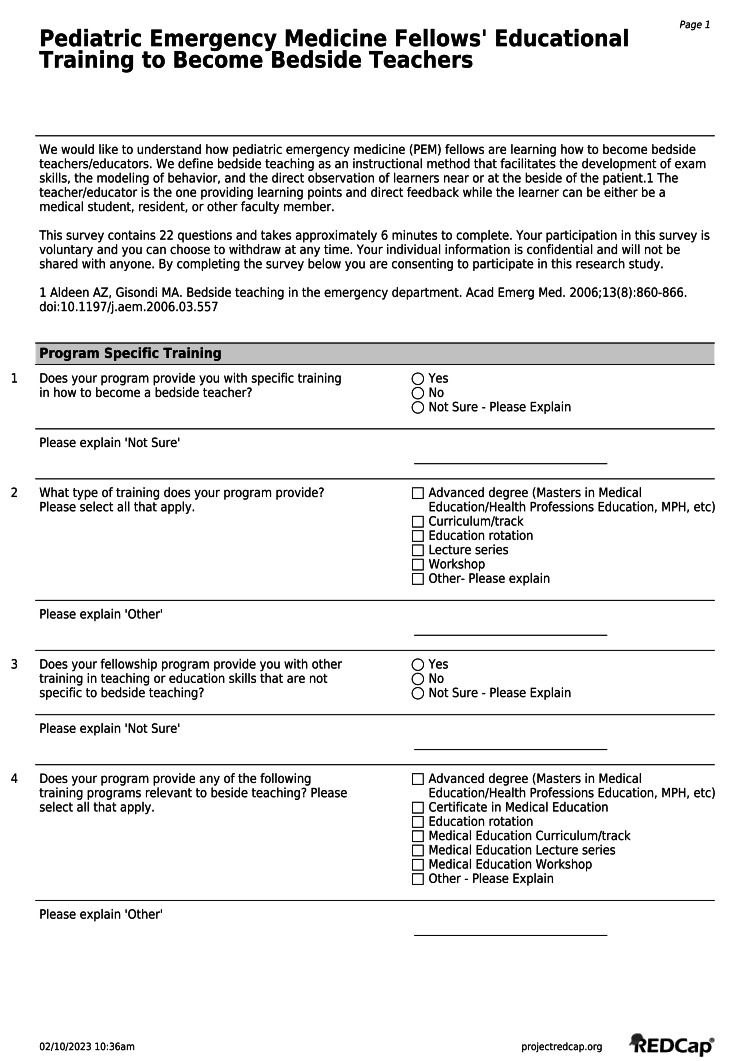
Fellow survey-page 1.

**Figure 2:**
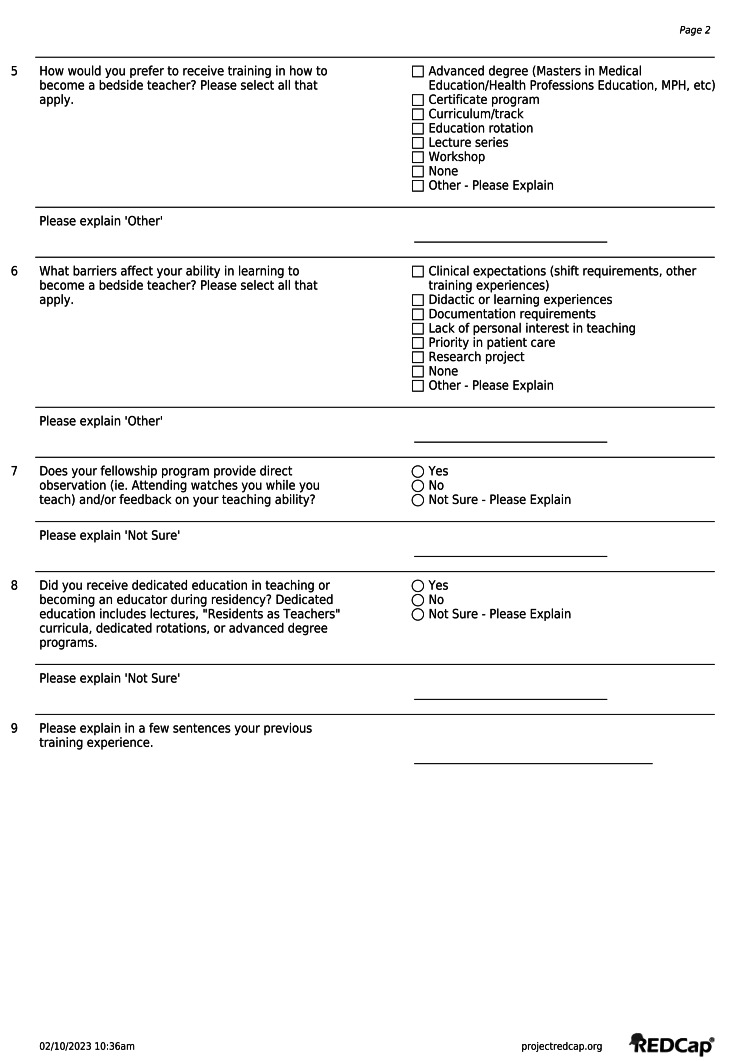
Fellow survey-page 2.

**Figure 3:**
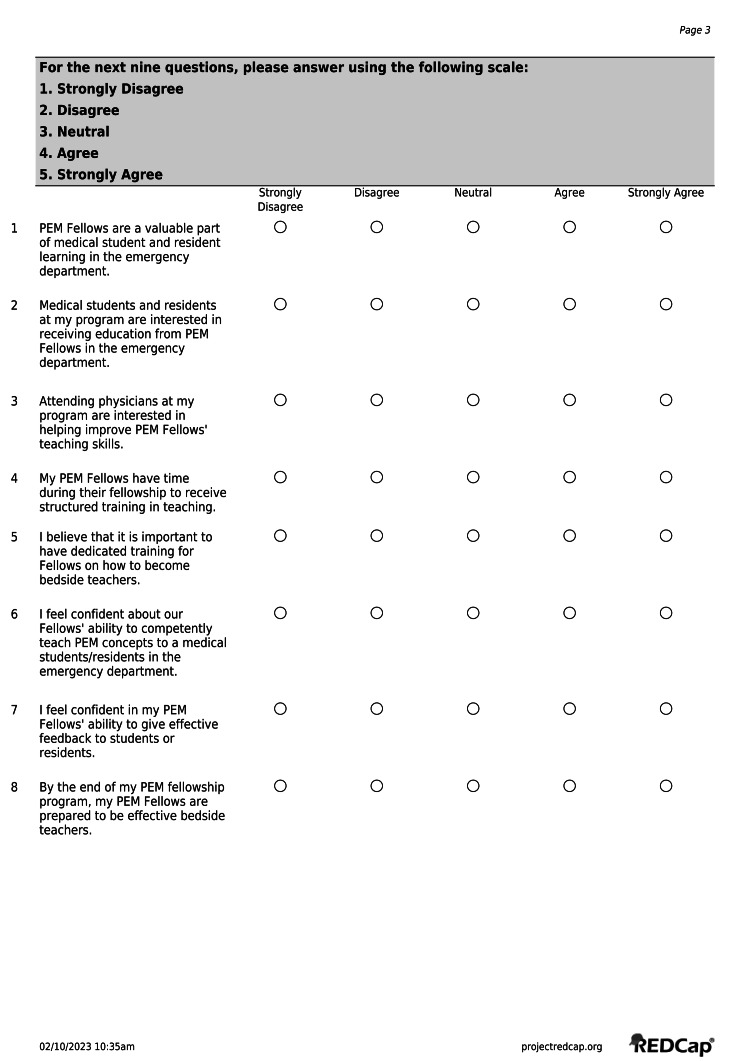
Fellow survey-page 3.

**Figure 4:**
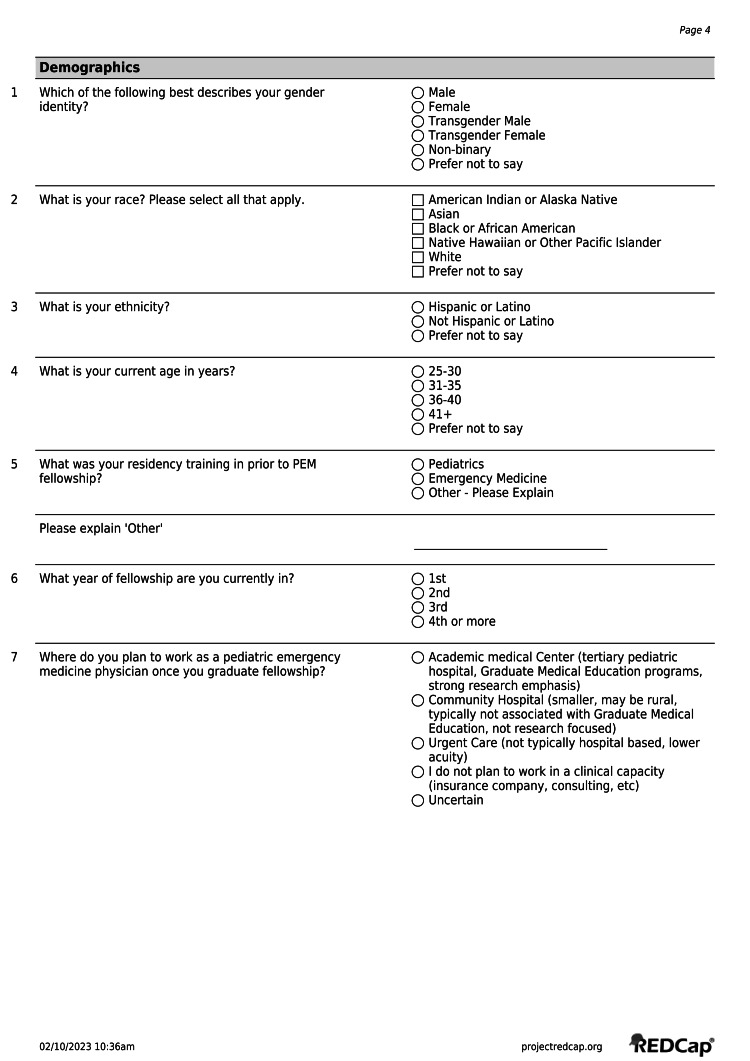
Fellow survey-page 4.

**Figure 5:**
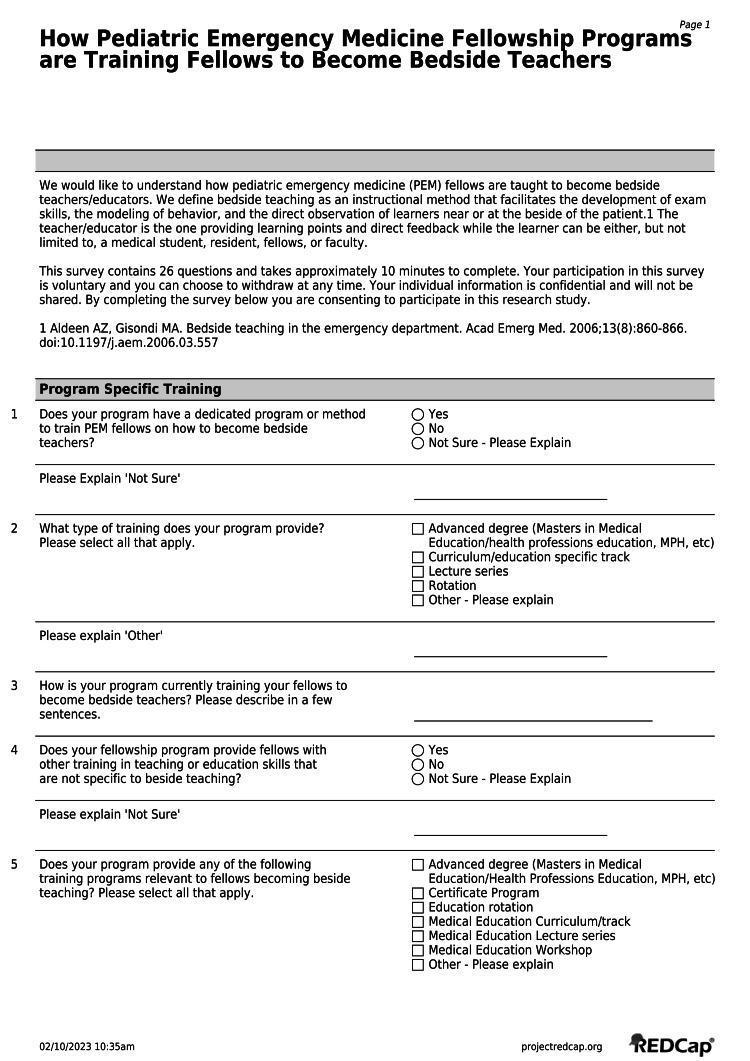
PD/APD survey-page 1. PD: program director, APD: associate program director.

**Figure 6:**
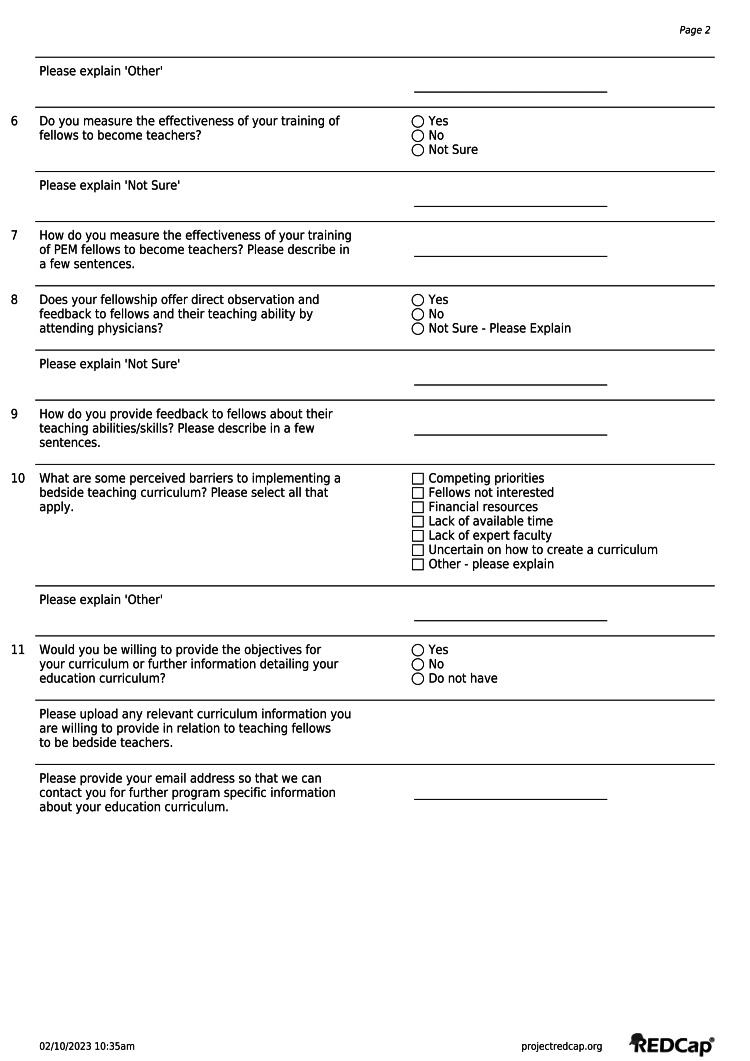
PD/APD survey-page 2. PD: program director, APD: associate program director.

**Figure 7:**
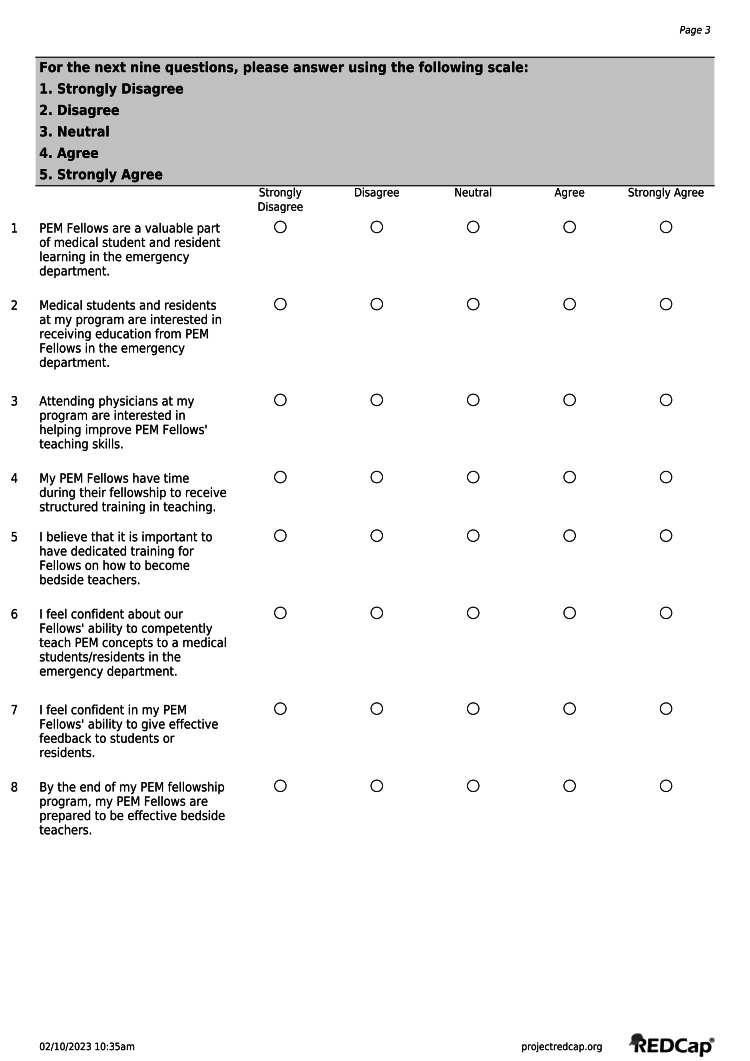
PD/APD survey-page 3. PD: program director, APD: associate program director.

**Figure 8:**
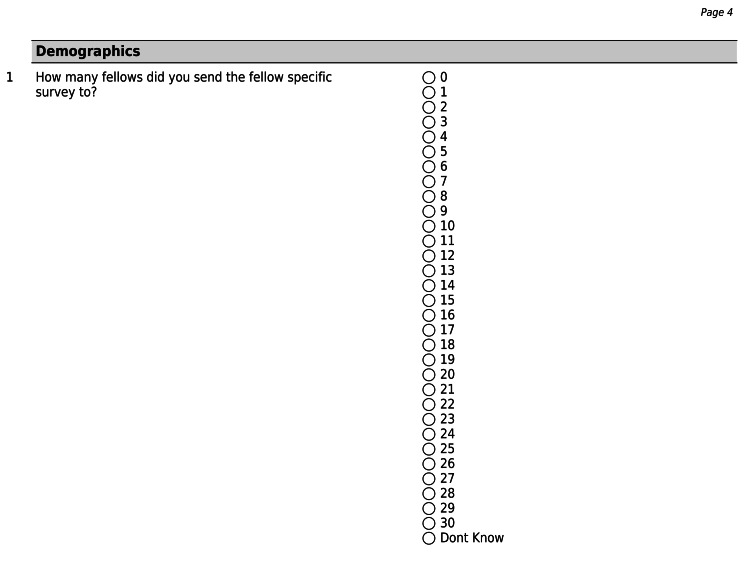
PD/APD survey-page 4. PD: program director, APD: associate program director.

**Figure 9:**
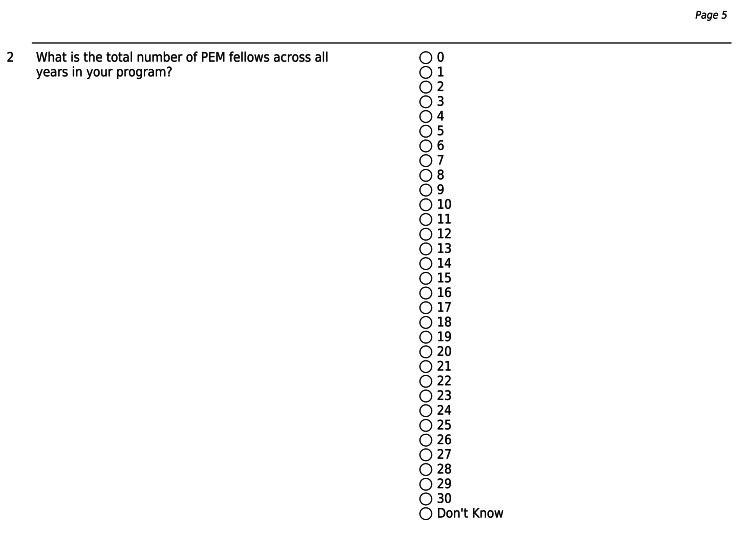
PD/APD survey-page 5. PD: program director, APD: associate program director.

**Figure 10:**
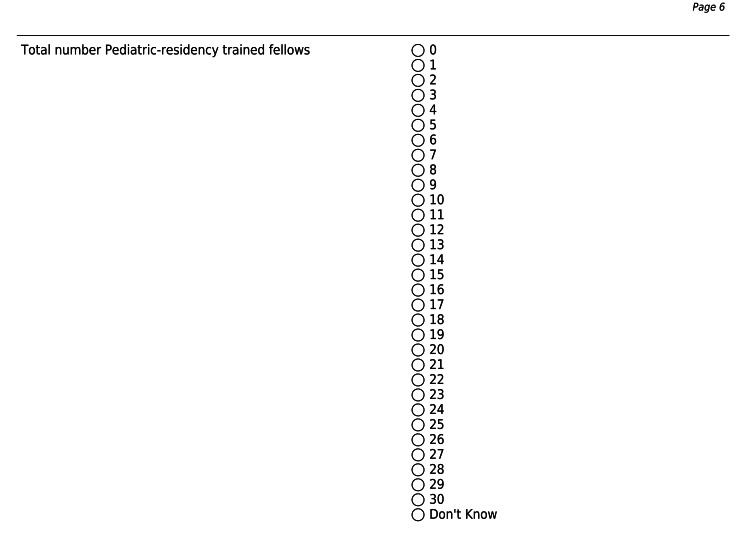
PD/APD survey-page 6. PD: program director, APD: associate program director.

**Figure 11:**
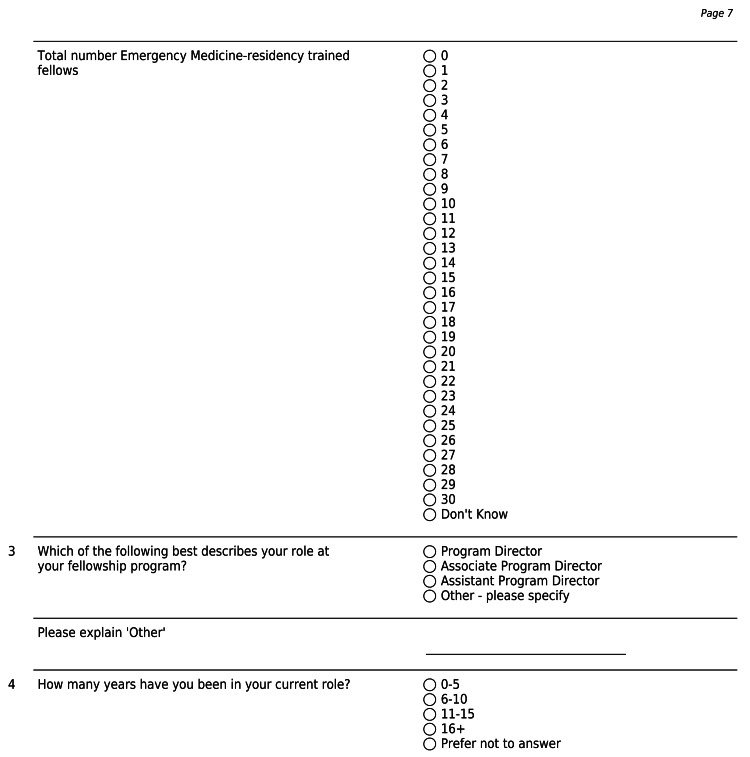
PD/APD survey-page 7. PD: program director, APD: associate program director.

**Figure 12:**
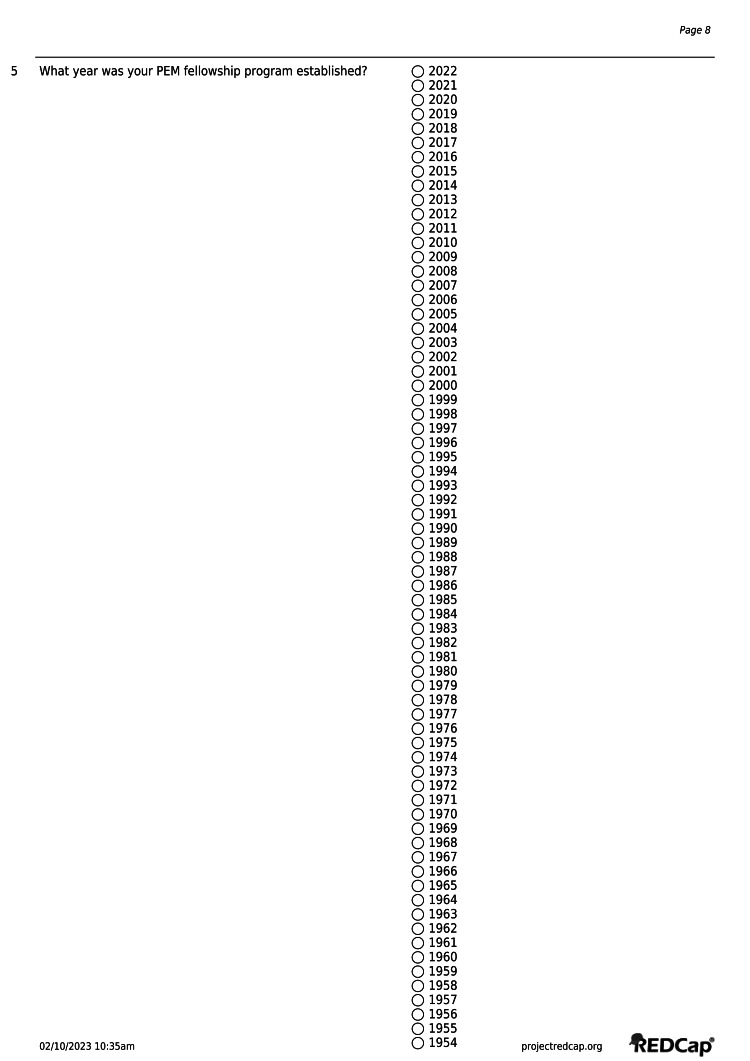
PD/APD survey-page 8. PD: program director, APD: associate program director.

**Figure 13:**
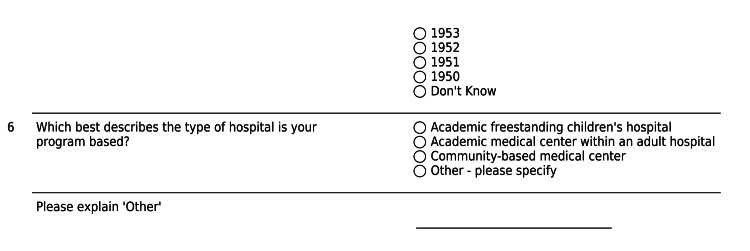
PD/APD survey-page 9. PD: program director, APD: associate program director.

We created two separate anonymous surveys: one for PEM fellows and one for PEM PDs/APDs. The majority of questions were developed independently, but a few questions were adapted from previously conducted surveys [14]. PEM fellows completed a 20-question survey that assessed their program’s training in bedside teaching and gathered information about their perceptions of their current training and readiness to serve as bedside teachers upon graduation (see Appendix, Figures 1-4). PEM PDs/APDs completed a 23-question survey to determine if their respective PEM fellowship programs had a dedicated bedside teaching curriculum (see Appendix, Figures 5-13). If the respondents answered ‘yes’ to having a dedicated curriculum, they were then queried about the specific methods used in training fellows and assessing curricular effectiveness. We collected demographic data for both the fellows and PDs/APDs. The survey was piloted among non-PEM fellows and non-PEM PDs at the study institution to gather feedback on the survey questions and to ensure understanding.

We contacted PEM fellows and PDs/APDs via email through the American Academy of Pediatrics Section of Emergency Medicine PEM PD Survey Committee. This committee, operating at the national level, reviews potential surveys from PEM fellows/PDs and disseminates these surveys directly to all PDs of fellowship programs. Subsequently, the PEM PDs electronically forwarded the survey to their respective PEM fellows. The PEM PD Survey Committee sent two survey email reminders to PDs to encourage them to remind their respective PEM fellows. Given the survey dissemination method established by the PEM PD Survey Committee, we were unable to directly track the number of fellows and PDs/APDs who received the survey invitation and whether they responded. We used REDCap™ (Research Electronic Data Capture) for survey development and data collection [17]. 

Analysis

Tabulations, test results, and figures were produced by R Statistical Software (v4.1.0; R Core Team 2021) [18]. Demographics for fellows and programs were summarized as n (%) for categorical variables and median (IQR) for numerical variables. Survey responses that were originally based on a five-point Likert scale were collapsed into three categories before analysis: “Agree” (“Strongly Agree” and “Agree”), “Neutral," and "Disagree" ("Disagree" and "Strongly Disagree"). Relationships between categorical variables in contingency tables were analyzed using Fisher's exact tests. Results with p-values <0.05 were considered to be statistically significant. Additional contingency tables were built to explore joint distributions between variables without formal statistical testing.

## Results

Survey participants

PDs/APDs were asked how many fellows they forwarded the survey to. Based on their responses, a total of 211 PEM fellows received the survey, of which 69 fellows completed it (response rate 32.7%, n=69/211). We requested that only one PD or APD complete the survey from each PEM fellowship program. Out of the 80 PEM programs in total, 21 PDs and five APDs completed the survey, with the expectation that one individual from each program would complete the survey (response rate 32.5%, n=26/80). Table 1 shows the demographic data of the survey respondents.

**Table 1:** Demographics among pediatric emergency medicine fellow and program director and associate program director study respondents. ^a^Tertiary pediatric hospital, Graduate Medical Education programs, strong research emphasis. ^b^Smaller, may be rural, typically not associated with Graduate Medical Education, not research-focused. ^c^Not typically hospital-based, lower acuity. ^d^Insurance company, consulting, etc.

Demographics of respondents	n (%)
Fellow demographics	
Gender	
Male	18 (26.1)
Female	51 (73.9)
Age (years)	
25-30	10 (14.5)
31-35	49 (71)
36-40	6 (8.7)
41+	1 (1.4)
Prefer not to say	3 (4.3)
Residency training prior to pediatric emergency medicine fellowship	
Pediatrics	65 (94.2)
Emergency medicine	3 (4.3)
Other	1 (1.4)
Year of fellowship	
1st	28 (40.6)
2nd	16 (23.2)
3rd	22 (31.9)
4th or more	3 (4.3)
Post-graduation work plans	
Academic medical center^a^	52 (75.4)
Community hospital^b^	6 (8.7)
Urgent care^c^	1 (1.4)
I do not plan to work in a clinical capacity^d^	2 (2.9)
Uncertain	8 (11.6)
Program directors/associate program directors demographics	
Role at fellowship program	
Program director	21 (80.8)
Associate program director	5 (19.2)
Assistant program director	0 (0)
Years in your current role	
0-5	15 (57.7)
6-10	5 (19.2)
11-15	5 (19.2)
16+	1 (3.8)
Type of hospital	
Academic freestanding children's hospital	17 (65.4)
Children’s hospital within an adult hospital	8 (30.8)
Community-based medical center	1 (3.8)

PEM fellow survey results

Most respondents completed a pediatrics residency (94.2%; n=65/69) prior to starting a PEM fellowship. More than half of PEM fellows (59.4%, n=41/69) stated that their program did not offer specific bedside teaching curricula. However, almost all fellows (95.7%, n=66/69) were interested in bedside teaching education. Nearly two-thirds of PEM fellows (61%, n=42/69) reported that their program provided other training in teaching, such as advanced degrees (31.9%, n=22/69), lecture series (30.4%, n=21/69), and workshops (18.8%, n=13/69) (Table 2). The majority of PEM fellows (91.3%, n=63/69) felt that they were a valuable part of trainee learning in the emergency department.

**Table 2:** Frequency of other education training opportunities provided to fellow respondents.

	Yes	No	Not sure
Question from the fellow survey	n (%)	n (%)	n (%)
Does your fellowship program provide you with other training in teaching or education skills that are not specific to bedside teaching?	42 (60.9)	25 (36.2)	2 (2.9)
Does your fellowship program provide direct observation (i.e., attending watches you while you teach) and/or feedback on your teaching ability?	38 (55.1)	26 (37.7)	5 (7.2)
Did you receive dedicated education in teaching or becoming an educator during residency? Dedicated education includes lectures, ‘Residents as Teachers’ curricula, dedicated rotations, or advanced degree programs	43 (62.3)	25 (36.2)	1 (1.4)

Most fellows believed barriers to learning bedside teaching included clinical expectations (60.9%, n=42/69), patient care prioritization (56.5%, n=39/69), and documentation requirements (40.6%, n=28/69). The remaining barriers are summarized in Table 3. For the preferred form of training in bedside teaching, fellows expressed a preference for workshops (56.5%, n=39/69), dedicated curricula (42%, n=29/69), or dedicated rotations (40.6%, n=28/69).

**Table 3:** Perceived barriers to learning bedside teaching from fellow and program director/associate program director respondents. ^a^Fellows were able to select all barriers that apply. Barriers are listed in the order presented in the survey. ^b^Program directors were able to select all barriers that apply. Barriers are listed in the order presented in the surveys.

	n (%)
What barriers affect your ability in learning to become a bedside teacher?^a^	
Clinical expectations (shift requirements, other training experiences)	42 (60.9)
Didactic or learning experiences	12 (17.4)
Documentation requirements	28 (40.6)
Lack of personal interest in teaching	3 (4.3)
Priority in patient care	39 (56.5)
Research project	13 (18.8)
None	17 (24.6)
What are some perceived barriers to implementing a bedside teaching curriculum?^b^	
Competing priorities	16 (61.5)
Fellows not interested	2 (7.7)
Financial resources	4 (15.4)
Lack of available time	16 (61.5)
Lack of expert faculty	7 (26.9)
Uncertain how to create a curriculum	14 (53.8)

Three-quarters of fellows (75.4%, n=52/69) expressed interest in working at an academic medical center upon graduation. Among the fellows who expressed interest in working at an academic medical center after training, 65.4% (n=34/52) reported that their current programs do not have formal training in bedside teaching. Additionally, 19.2% (n=10/52) of fellows felt unprepared for bedside teaching by the end of the fellowship. No significant association was found between the year of fellowship training and the perception of preparedness to be a bedside teacher (p=0.07). Similarly, there was no significant association between whether a program had formal bedside teaching training and the fellows’ perceptions of programmatic effectiveness in preparing them to be bedside teachers (p=0.40).

PD/APD survey results

Of the PD/APDs who completed the survey, 76.9% (n=20/26) indicated that their program did not have a dedicated bedside teaching curriculum. More than two-thirds of PDs/APDs (69.2%, n=18/26) reported that their fellows received additional training in teaching skills during their PEM fellowship. This additional training included advanced degrees (38.5%, n=10/26), lecture series (38.5%, n=10/26), and workshops (26.9%, n=7/26). Additionally, over half of PDs/APDs (61.5%, n=16/26) noted that competing priorities of fellows and a lack of available time were barriers to implementing a bedside teaching curriculum (Table 3).

All PDs/APDs agreed that fellows have a valuable role in the education of medical students and residents in the PED. The majority of PDs/APDs (80.8%, n=21/26) stated they provided some form of observation and feedback to fellows on their teaching skills. Also, 92.3% (n=24/26) agreed that faculty at their program are interested in helping fellows in improving their teaching skills. Table 4 summarizes PDs’/APDs’ perceptions of their local training environment.

**Table 4:** Frequencies of fellow and program director/associate program director respondents’ perceptions of their local training environment.

	Strongly disagree	Disagree	Neutral	Agree	Strongly agree
	n (%)	n (%)	n (%)	n (%)	n (%)
Fellows' survey items					
I enjoy teaching medical students and residents.	0	1 (1.4)	4 (5.8)	28 (40.6)	36 (52.2)
Students and residents at my program are interested in receiving education from Pediatric Emergency Medicine Fellows when they are in the emergency department.	0	0	6 (8.7)	36 (52.2)	27 (39.1)
Fellows are a valuable part of medical student and resident learning in the emergency department.	0	0	6 (8.7)	19 (27.5)	44 (63.8)
Faculties at my Pediatric Emergency Medicine fellowship program are interested in helping me improve my teaching skills.	0	2 (2.9)	18 (26.1)	34 (49.3)	15 (21.7)
I think that it is important to have dedicated training on how to become a bedside teacher.	1 (1.4)	2 (2.9)	11 (15.9)	36 (52.2)	19 (27.5)
I feel confident in my ability to teach Pediatric Emergency Medicine to residents and medical students.	0	1 (1.4)	16 (23.2)	35 (50.7)	17 (24.6)
At the end of my Pediatric Emergency Medicine fellowship, I feel/will feel prepared to be a bedside teacher.	0	1 (1.4)	12 (17.4)	28 (40.6)	28 (40.6)
I enjoy teaching medical students and residents.	0	1 (1.4)	4 (5.8)	28 (40.6)	36 (52.2)
Program directors'/associate program directors' survey items					
Pediatric Emergency Medicine Fellows are a valuable part of medical student and resident learning in the emergency department.	0	0	0	5 (19.2)	21 (80.8)
Medical students and residents at my program are interested in receiving education from Pediatric Emergency Medicine Fellows in the emergency department.	0	0	2 (7.7)	6 (23.1)	18 (69.2)
Attending physicians at my program are interested in helping improve Pediatric Emergency Medicine Fellows’ teaching skills.	0	0	2 (7.7)	15 (57.7)	9 (34.6)
My Pediatric Emergency Medicine Fellows have time during their fellowship to receive structured training in teaching.	0	4 (15.4)	7 (26.9)	6 (23.1)	9 (34.6)
I believe that it is important to have dedicated training for fellows on how to become bedside teachers.	0	2 (7.7)	4 (15.4)	12 (46.2)	8 (30.8)
I feel confident about our fellows’ ability to competently teach Pediatric Emergency Medicine concepts to medical students/residents in the emergency department.	0	0	2 (7.7)	12 (46.2)	12 (46.2)
I feel confident in my Pediatric Emergency Medicine Fellows’ ability to give effective feedback to students or residents.	0	3 (11.5)	5 (19.2)	12 (46.2)	6 (23.1)
By the end of my Pediatric Emergency Medicine fellowship program, my fellows are prepared to be effective bedside teachers.	0	0	0	17 (65.4)	9 (34.6)

Less than one-fifth (19.2%, n=5/26) of PDs/APDs reported measuring the effectiveness of their bedside teaching curriculum. Among those who did measure curricular effectiveness, assessments were conducted through evaluations (60%, n=3/5), direct feedback (20%, n=1/5) and direct observations (20%, n=1/5). Over half of PDs/APDs (53.8%, n=14/26) expressed that they did not know how to create a dedicated bedside teaching curriculum.

Comparison of PEM fellow and PD/APD results

Of the respondents, 81.2% (n=56/69) of fellows believed that their programs prepared them to be effective bedside teachers, whereas 100% of PDs/APDs shared this belief (p-value=0.03). Furthermore, 75.4% (n=52/69) of fellows stated they felt confident in their ability to teach PEM to residents and medical students, while 92.3% of PDs/APDs were confident in their fellows’ abilities (p-value=0.086).

## Discussion

Most PEM fellowship programs lacked formal curricula in bedside teaching. Additionally, nearly one-fifth of PEM fellows felt unprepared to be bedside teachers upon graduation, highlighting the potential need for dedicated curricula in bedside education. Furthermore, one-quarter reported not feeling confident in their ability to teach PEM to trainees. A formal teaching curriculum has been shown to improve fellows’ confidence to teach learners and could address both concerns [9,10].

While most fellows felt adequately prepared for bedside teaching upon graduation, they still emphasized the importance of having a dedicated bedside teaching curriculum. This desire for dedicated bedside teaching education may arise from the fact that the majority of surveyed PEM fellows expressed a strong interest in pursuing careers in academic institutions after graduation, where the expectation is to provide bedside instruction to trainees [8]. Even within programs that provided formal bedside teaching education, the absence of methods to evaluate curricular effectiveness could raise concerns about the adequacy of the training provided to fellows in bedside teaching. This may also help explain the lack of a significant association between whether a program provided formal training in bedside teaching and how fellows perceived the effectiveness of this training in preparing them to become bedside teachers. Furthermore, the lack of curricular evaluation might explain the overconfidence of PDs/APDs in their fellow’s preparedness to be bedside teachers upon graduation [19]. Therefore, addressing this discordance may be best achieved through the implementation of formal bedside teaching curricula. 

Our study found that the majority of responding PDs/APDs were uncertain on how to develop and create content for a dedicated bedside teaching curriculum. Well-known medical education curriculum development strategies include the following six steps: 1) performing a needs assessment, 2) determining content, 3) writing goals and objectives, 4) selecting educational delivery strategies, 5) implementing the curriculum, and 6) evaluating the curriculum [19]. The results from this study serve as the initial needs assessment.

To determine curriculum content, the Council of Emergency Medicine Residency Directors suggests incorporating training into various teaching models, along with the use of simulation and just-in-time training technologies to enhance teaching skills [12]. The curriculum’s goals should be broad to address the overall need, while the objectives should be specific, measurable, attainable, relevant, and time-bound (SMART) [20]. Evaluating the curriculum by learners is essential to ensure the achievement of goals and objectives, and the evaluation of trainees is equally crucial. According to the ACGME, an ideal assessment of a trainee should determine and guide their developmental progression while providing informative feedback [21]. Many of the aforementioned references are available at no cost and can provide a framework for initial curriculum development.

PEM fellowship programs will also have to balance competing priorities during curriculum implementation. In this study, both fellows and PDs/APDs expressed that other clinical expectations were barriers to bedside teaching. Specifically, trainees and attendees expressed concern that taking time to teach can impact clinical productivity [2,4]. However, teaching trainees can actually enhance patient throughput in the ED [22,23]. The presence of trainees in the emergency department has also been shown to decrease the number of patients who leave without being seen [24]. In addition, teaching methods such as the one-minute preceptor, a learner-centered model based on what they think is going on with the patient and probing for supporting evidence, allow for information dissemination in bite-sized pieces without impacting patient care [25]. Emergency Department Strategies for Teaching Any Time (ED STAT) is another tool that incorporates clinical teaching and feedback into a single model, making it an efficient technique in the ED [12]. In a time when emergency department patient volumes are increasing, formal education on efficient and effective bedside teaching skills will not only benefit trainees but also positively impact patients.

Limitations

This study has limitations. The survey response rate was relatively low, which can be attributed to our inability to directly follow up with non-responders. This limitation arises from the survey’s anonymous dissemination method through the PEM PD Survey Committee, which was implemented to protect responders’ anonymity. Additionally, given our study design, we did not capture the reflections of new PEM fellow graduates, which would provide insight into fellowship gaps in training. Future research is warranted to explore the impact that bedside teaching curricula would have on trainees and the PED. 

## Conclusions

In the unique environment of a busy emergency medicine department, PEM fellows must be trained to become efficient and effective bedside teachers. Our study underscores the necessity for a dedicated curriculum tailored specifically for bedside teaching PEM fellowships. Given the significant interest among PEM fellows in pursuing careers as educators and most PEM programs lacking a dedicated approach to training fellows in bedside teaching, future endeavors may focus on formalizing such education and targeted curriculum development.
